# Sharing Wisdom, Sharing Hope: Strategies Used by Native American Cancer Survivors to Restore Quality of Life

**DOI:** 10.1200/JGO.19.00215

**Published:** 2020-01-13

**Authors:** Tiana D. Bastian, Linda Burhansstipanov

**Affiliations:** ^1^ Professional Data Analysts, Minneapolis, MN; ^2^ Native American Cancer Research Corporation, Pine, CO

## Abstract

**PURPOSE:**

The purpose of this study was to gain insight into the experiences of Native American cancer survivors in navigating life after cancer and what resources and strategies survivors found useful for coping and achieving optimal quality of life (QoL) after diagnosis (the terms “Native Americans” and “Natives” are used interchangeably in this article to describe American Indians and Alaska Natives). The research questions were What advice and words of wisdom do Native cancer survivors prioritize in messages to other Native cancer survivors? and What do those messages reveal about how Native cancer survivors interpret, experience, and restore QoL after diagnosis?

**METHODS:**

This study used a qualitative phenomenologic descriptive study design. Researchers used thematic analysis to identify themes related to peer advice and QoL from transcripts of semi-structured interviews with 52 geographically and clinically diverse Native cancer survivors in the United States.

**RESULTS:**

Survivors’ lived experiences directly informed their advice to other survivors, which was characterized by four themes: listen to your body, advocate for yourself, embrace your culture and spirituality, and share your story. A deeper look into the origins of those messages revealed challenges survivors face balancing their responsibility to care for themselves while simultaneously embracing cultural values of selflessness.

**CONCLUSION:**

Providers and researchers should work with Native cancer survivors to identify and leverage existing community strengths in ways that support all aspects of a survivors’ QoL rather than limiting support to a single QoL domain (eg, physical, spiritual, mental/emotional, or social issues). Interventions should ensure that supports and services align with survivors’ cultural values and attend to competing responsibilities to optimize QoL.

## INTRODUCTION

Native Americans have the poorest 5-year relative survival for all cancers combined (60%) and are more likely to die of their cancer than patients who identify as white in the United States.^1,2^ (The terms “Native Americans” and “Natives” are used interchangeably in this article to describe American Indians and Alaska Natives.) Statistics on cancer incidence, mortality, and survival provide estimates of the number of Natives diagnosed with cancer and length of survival, but they are not informative for estimating the impact of diagnosis on survivors’ daily lives, their families, and communities. In this article, a cancer survivor is defined as any individual diagnosed with cancer, from the time of diagnosis and for the balance of life.^3,4^ The four domains of survivorship (physical, psychological, social, and spiritual) described by Ferrell et al^5^ in 1997 continue to be essential measures of quality of life (QoL).^5-8^ Although QoL research among cancer survivors increased over the past decade, few studies focus on Native American cancer survivors.^9^ Of studies that have focused on Native cancer survivors, many are limited to single method studies, or they assess QoL domains independently, thus providing only a partial picture of QoL.^10-17^ Quantitative studies exclude survivor voices that could put data into context, and qualitative studies are not suited for estimating prevalence of or interaction between themes. This study composes the qualitative strand of a larger mixed methods study to address this research gap by using a mixed methods research approach to generate a holistic understanding of factors that influence QoL among Native cancer survivors and how those factors manifest in survivors’ daily lives.^18^ This study examined Native cancer survivors’ experiences navigating life after cancer and the strategies they found useful for restoring QoL after diagnosis through the lens of advice giving. Within cancer support networks, advice giving commonly reflects survivors’ personal experiences.^19^

**CONTEXT**

**Key Objective**
The purposes of this study were to honor study participants’ intentions to help other Native cancer survivors by sharing their cancer stories and to gain insight into the resources and strategies survivors found useful for coping and restoring QoL after diagnosis.
**Knowledge Generated**
This study answers the research questions, What advice and words of wisdom do Native cancer survivors prioritize in messages to other Native cancer survivors? and What do those messages reveal about how Native survivors interpret, experience, and restore QoL after diagnosis?
**Relevance**
Knowing what matters most to survivors for living well after cancer will help caregivers and researchers align supports with survivors’ preferences and needs.

## METHODS

This study used a descriptive phenomenologic research approach^20^ to describe the lived experience of Native American cancer survivors. Data for this study were de-identified verbatim transcripts from one-on-one semi-structured interviews with a geographically and clinically diverse convenience sample of 79 Native cancer survivors. Interviews were conducted by Native American Cancer Research Corporation (NACR) (http://natamcancer.org/) between 1994 and 2015. Participants consented to the interviews and to NACR using their interviews for research purposes. Twenty-seven transcripts were excluded from this qualitative, secondary data analysis because participants did not meet key inclusion criteria, including residence in the contiguous United States, being 18 years of age or older, and having ever received a diagnosis of cancer. A total of 52 transcripts were included in the analysis. A semi-structured interview protocol led survivors to narrate their cancer story in chronological order from diagnosis, to treatment, to recovery, and finally to giving advice to other survivors based on their own hindsight.

Authors used thematic analysis^21^ to identify themes in peer advice and ways in which that advice reflected survivors’ experiences restoring QoL after diagnosis. Themes were generated on the basis of survivors’ own words without attempting to interpret underlying ideas from participants’ narratives. NVivo (version 12) software was used to organize, code, and analyze the data. The original study was approved by the Colorado Multiple Institutional Review Board, and the current study was reviewed and determined exempt by the University of Minnesota Institutional Review Board.

## FINDINGS

Participant demographics are provided in Table 1. Just over half the survivors in the sample were breast cancer survivors, primarily because NACR survivorship programs began as programs for breast cancer survivors and only opened up to other cancer types in 1999.

**TABLE 1:**
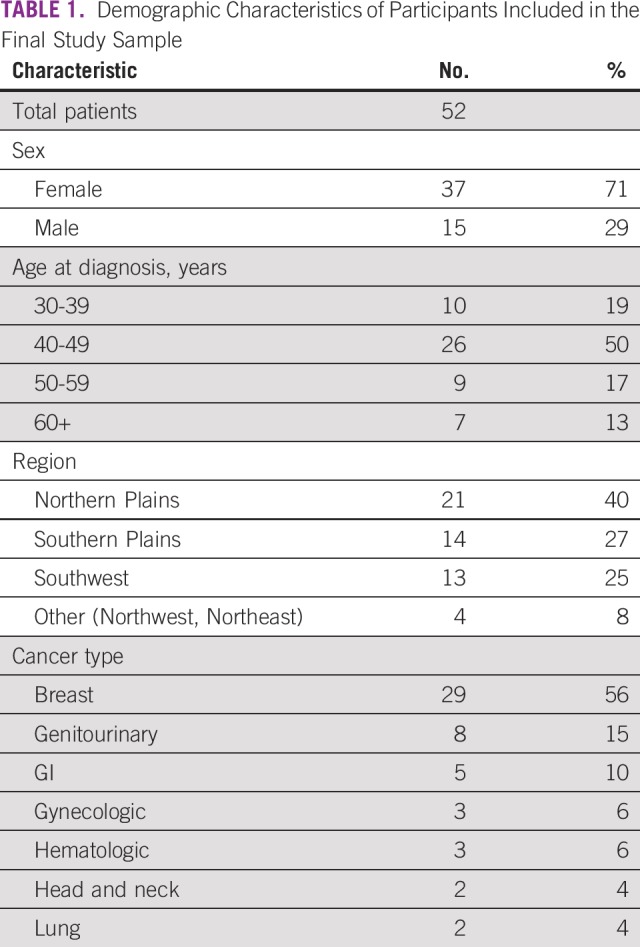
Demographic Characteristics of Participants Included in the Final Study Sample

A central theme of navigating life after cancer was balancing multiple responsibilities between self, family, and community. Survivors’ cancer experiences directly informed their advice to other Native cancer survivors, which are characterized by four overarching themes: listen to your body, advocate for yourself, embrace your culture and spirituality, and share your story.

### Theme 1: Listen to Your Body

This theme encourages Natives to recognize and trust their bodies to alert them when they are out of balance. This advice often reflected survivors’ personal experiences of becoming aware of their own illness and consequences of having acted or not acted on those messages. For many survivors, their first suspicion that something was wrong came from a general feeling of being out of harmony and balance. One survivor said,

I knew there was something wrong. The feelings I was getting were like a big void over my heart. And there was no pain. There was no life. It was nothing. It was something that was consuming my entire being. This was what the scary part was. That’s why I knew it was cancer before I was diagnosed. (Southwest, female, breast cancer)

Survivors emphasized, “We know our bodies better than anybody else, so listen to your body and trust it.” Among survivors who neglected to act on their body’s warning messages, many did so out of fear and avoidance. One survivor explained, “many times, Native Americans like myself wait, and wait, thinking, ‘well if I don’t go, it might go away.’” In addition to being able to communicate messages of illness, survivors also described the body as a powerful source of healing. Survivors encouraged other Natives to “have that inner faith in yourself, let your body help you heal yourself.”

### Theme 2: Advocate for Yourself

This theme describes the imperative for cancer survivors to take an active role in their care and healing in their personal life and in the health care setting. Underneath survivors’ advice to advocate for one’s own needs was an understanding that advocacy begins with believing you will survive. One survivor stated, “we live on hope.” Self-pity was described as something that “weakens you” and to be avoided at all costs. However, many survivors faced challenges accessing resources they needed for support and healing.

One challenge voiced by many female survivors was trying to take care of themselves while simultaneously fulfilling their responsibilities to care for others. One survivor described it as being a “very uncomfortable position when you have to start taking care of yourself.” Another survivor explained,

As Indian women, we have a tendency to make sure that everyone is doing well and we’re not interfering with them and they’re not worrying too much about us. So what happens is we don’t process the sadness because there is a loss … there is a loss of innocence when you’ve had cancer and you need to go through those feelings and process them. (Northern Plains, female, breast cancer)

Survivors felt responsible to continue being “the strong one” despite their limited capacity to do so in light of their cancer. These challenges made it difficult for survivors to ask for help or allow others to care for them. Sometimes putting the needs of others first came at a cost to survivors such as delayed diagnosis and treatment. One strategy for reconciling their dual responsibilities to self and others was by reasoning that taking care of themselves was a means of taking care of others.

Survivors’ discussed positive and negative experiences with the health care system, both of which informed their advice to other survivors to “be your own advocate.” Survivors emphasized the importance of taking someone with you to the doctor, asking questions, and getting second opinions. Knowledge was seen as a resource for communicating effectively with health care providers, calming fears about treatment, and easing the stress of decision making. One survivor shared,

What I needed to do was educate myself and figure out what exactly I was up against so I could formulate my battle plan. I armed myself with as much knowledge as I could find on the internet [and] talked with cancer survivors…Once I realized what I was up against, it was much easier for me to tackle it. (Northeast, female, breast cancer)

Education played an important role in facilitating survivors’ ability to advocate for their health care needs. Having knowledge about their cancer and treatment options helped survivors to ask the right questions, clarify expectations, and make informed decisions, all of which helped survivors feel prepared for the road ahead. In addition, educational attainment of survivors and/or their caregivers provided a pathway to employment and technical skills that survivors and/or caregivers drew upon to access health insurance, navigate the health system, get second opinions, research their cancer and treatment options, and fight to get appointments when they were not being heard.

### Theme 3: Embrace Your Culture and Spirituality

Feeling connected to Native people and culture was a source of strength and grounding for many survivors. Survivors described the importance of “keep[ing] your roots with your people, to learn who you are and where you come from.” Some survivors, who felt disconnected from Native people and culture before their cancer diagnosis, returned to the reservation or took steps to learn about and participate in cultural ceremonies after their cancer diagnosis. Nearly all survivors reflected on the prominence of spirituality as a source of hope and comfort during all stages of their cancer journey.

Putting one’s trust in the Creator and embracing life as a precious “one-time gift given to us by a higher power” allowed survivors to reframe ostensibly negative experiences through a positive lens of gratitude. One survivor reflected,

I was so privileged, I guess it’s a bad thing to say, privileged to having cancer. It just helped me a lot to reach out to others and not be centered on myself. (Southern Plains, female, non-Hodgkin lymphoma)

Survivors emphasized the limitations of Western medicine, which focuses on treating the body while neglecting the spirit and mind. This narrow approach to healing does not align with a Native perspective of health, which survivors described as “holistic” and being in “balance” and “harmony.” Healing from cancer meant “living both ways” by embracing Western medicine and traditional healing strategies. One survivor explained,

When you walked into the doctor’s office and he told you he’s going to take something out of you, you can darn well bet he took part of your spirit from you. It was like a gasp of air that rushed out of me, I felt the hole that he shot in me. And then I went to six or seven doctors and they all shot me full of holes, too. The spiritual part of closing those wounds is as important as the physical part. (Southwest, male, head and neck cancer)

### Theme 4: Share Your Story

At the heart of survivors’ interviews was a desire to help other Natives by sharing their cancer stories and subsequently, sharing hope. Sharing their cancer stories served two primary purposes: helping survivors cope with their own illness and inspiring hope among other Native cancer survivors. Feeling understood by family and friends was important to survivors’ QoL because it opened the door for them to talk about and process their emotions and cope with their illness. Many survivors credited their families for providing the encouragement, strength, and courage they needed to survive their cancer. Connecting with other cancer survivors was an especially important source of solace and support.

For many survivors, bearing witness to other Natives surviving and thriving after cancer was so powerful for inspiring hope that it became imperative for them to share their experience with others. Although witnessing others die of cancer served to justify survivors’ fears of cancer being a death sentence, witnessing others survive cancer provided hope for survival and inspiration for living. For some survivors, meeting other Natives who were surviving and thriving after cancer was a turning point in their cancer journey. One survivor shared her experience of attending a cancer support group saying,

When the meeting started, each one introduced themselves, [shared] what they had gone through, and how long they had been survivors. It was anywhere from one to forty years. I looked around and said, “They’re all survivors? If they can do it, I can do it!” (Southwest, female, bladder cancer)

Some survivors drew a connection between their cancer experiences and their life purpose believing in a purpose for everything, “even if it’s the purpose to serve in cancer research.” For one survivor it meant, “I had to celebrate the cancer, too, because it was there for a reason, and I had to learn something.” Notably, some survivors chose not to talk about their cancer because they did not want to be pitied, or they perceived their cancer as something in the past and just wanted to move on.

## DISCUSSION

This study reinforced findings from previous research of the central reinforcing roles that spirituality, family, community, and survivors themselves play in maintaining a positive QoL after a cancer diagnosis.^14,22-24^ When caring for Native cancer survivors, health care providers should seek to understand survivors’ competing responsibilities and not assume that survivors will prioritize their own needs over others. This study’s results concur with Braun et al^25^ that framing self-care as a strategy for caring for others may be a useful way to encourage Native cancer survivors to take actions needed to stay strong and well. Providers can also support survivors by providing opportunities to advocate for their needs, such as through patient navigation programs.^26^ Recommendations informed by this study include interventions to increase knowledge about cancer, encourage asking questions, provide access to second opinions, and encourage survivors to bring someone with them to appointments. Future studies should test the feasibility and clinical usefulness of including questions on QoL assessment tools that ask survivors how well they are balancing responsibilities between self and community, the extent to which they feel they are a burden to others, and how well their family is responding to their diagnosis.

Finding that survivors felt compelled to use their experience to help other survivors is consistent with previous research in which survivors described their cancer experience as a gift they could give to their communities.^22,25^ However, not all survivors chose to talk about their cancer experience with others. One consequence of this silence is the unintentional support of the prevalent narrative of cancer as a death sentence in some Native communities. This may prevent screening and early detection among people who are unaware of their cancer risk.^27^ This study’s findings suggest that providing opportunities for Native cancer survivors to share their cancer stories may support healing and serve as a powerful way of inspiring hope among Native cancer survivors and their communities.

In concordance with previous research, finding strength in one’s spirituality, Native identity, and traditions were critical to survivors’ cancer journeys and healing.^23,24,27^ Circumstances that prevent survivors from readily accessing family, community, spiritual, and cultural resources may have especially devastating effects on survivors’ QoL.^28^ Interventions to support Native cancer survivors should integrate spirituality and prioritize activities that maintain survivors’ connections to family, culture, and community.

Interpreting findings from this research requires consideration of study strengths and limitations. One limitation is that data were from a convenience sample, which may have biased findings toward positive cancer experiences. However, learning from survivors who are motivated and thriving after cancer provides an opportunity to learn from “positive deviance” and identifies contexts and strategies that supported these survivors to thrive despite a difficult experience of cancer.^29^ Another limitation is that this study did not engage in theoretical sampling, which may have compromised research quality. However, authors identified multiple overlapping themes between this study and the study by Krebs.^30^ The Krebs study analyzed interview transcripts from a subsample of NACR interviews from 15 breast cancer survivors from 1994 to 1999, which validated the quality of the interpretive analysis and study findings for more recent survivors (ie, 1999 through 2015). Finally, this study did not explore differences in themes by year of diagnosis although advancements in cancer treatment over the study period may have influenced the survivorship experience. Study findings may not reflect the experience of survivors diagnosed in more recent years. Future research might address these limitations by sampling more recently diagnosed cancer survivors (eg, diagnosed within the past 5 years) and survivors representing a range of QoL experiences (ie, thriving or experiencing QoL limitations) to compare findings with those in this study and learn from a wider range of experiences.

In conclusion, this study provided independent validation of previous QoL research among Native American cancer survivors. Survivors’ advice to their peers was distilled into four overarching themes: listen to your body, advocate for yourself, embrace your culture and spirituality, and share your story. A deeper investigation into the origins of those messages revealed challenges survivors face when balancing responsibilities to care for themselves while simultaneously embracing cultural values of selflessness. When working with Native patients to understand their support needs, providers and researchers should ask questions to help them understand survivors’ values and priorities, because this information may be more informative for prioritizing supports than information on health status or symptoms alone.

Study findings have many implications for supporting Native cancer survivors on their journey to healing, many of which align with previous studies, including providing opportunities for survivors to share their cancer story with other Natives,^31^ attending to spirituality in all aspects of support,^23^ and providing survivors and their families with knowledge, tools, and supports for self-advocacy.^12,28^ This research describes multiple strengths and resources that exist in Native communities, including a strong sense of identity and belonging, spirituality, family and community connectedness, and collective responsibility to care for each other. Population-level interventions that target social determinants of health, especially those developed as collaborations between Native communities and researchers, may foster environments that make it easier for communities to bolster their strengths in ways that support cancer survivors.^32,33^
